# MicroRNA-205-5p plays a suppressive role in the high-fat diet-induced atrial fibrosis through regulation of the EHMT2/IGFBP3 axis

**DOI:** 10.1186/s12263-022-00712-z

**Published:** 2022-07-20

**Authors:** Zezhou Xiao, Yu Xie, Fangze Huang, Jie Yang, Ximao Liu, Xuefeng Lin, Peng Zhu, Shaoyi Zheng

**Affiliations:** Department of Cardiovascular Surgery, Nanfang Hospital, Southern Medical University, Guangzhou, Guangdong 510515 People’s Republic of China

**Keywords:** MicroRNA-205-5p, EHMT2, IGFBP3, Atrial fibrosis, High-fat diet

## Abstract

**Objective:**

MicroRNAs (miRNAs) targeting has been revealed to be an appealing strategy for the treatment and management of atrial fibrillation (AF). In this research, we aimed to explore the mechanisms of miR-205-5p in reducing the high-fat diet (HFD)-induced atrial fibrosis through the EHMT2/IGFBP3 axis.

**Methods:**

Expression levels of miR-205-5p, IGFBP3 and EHMT2 were determined in AF patients, cell fibrosis models and mouse atrial fibrosis models. Luciferase activity and RIP assays were performed to detect the binding between miR-205-5p and EHMT2, and ChIP assays were implemented to detect the enrichment of H3K9me2 and H3K4me3 in the promoter region of IGFBP3 in cells. The related experiments focusing on the inflammatory response, atrial fibrosis, mitochondrial damage, and metabolic abnormalities were performed to figure out the roles of miR-205-5p, IGFBP3, and EHMT2 in cell and mouse atrial fibrosis models.

**Results:**

Low expression levels of miR-205-5p and IGFBP3 and a high expression of EHMT2 were found in AF patients, cell fibrosis models and mouse atrial fibrosis models. Upregulation of miR-205-5p reduced the expression of TGF-β1, α-SMA, Col III and other fibrosis-related proteins. miR-205-5p overexpression targeted EHMT2 to regulate the methylation of H3 histones to promote IGFBP3 expression, which in turn affected the fibrosis of atrial muscle cells. In HFD-induced atrial fibrosis mice, upregulated miR-205-5p or elevated IGFBP3 alleviated atrial fibrosis, mitochondrial damage, and metabolic abnormalities.

**Conclusion:**

This study suggests that miR-205-5p attenuates HFD-induced atrial fibrosis via modulating the EHMT2/IGFBP3 axis.

**Graphical Abstract:**

miR-205-5p alleviates high-fat diet-induced atrial fibrosis in mice via EHMT2/IGFBP3.

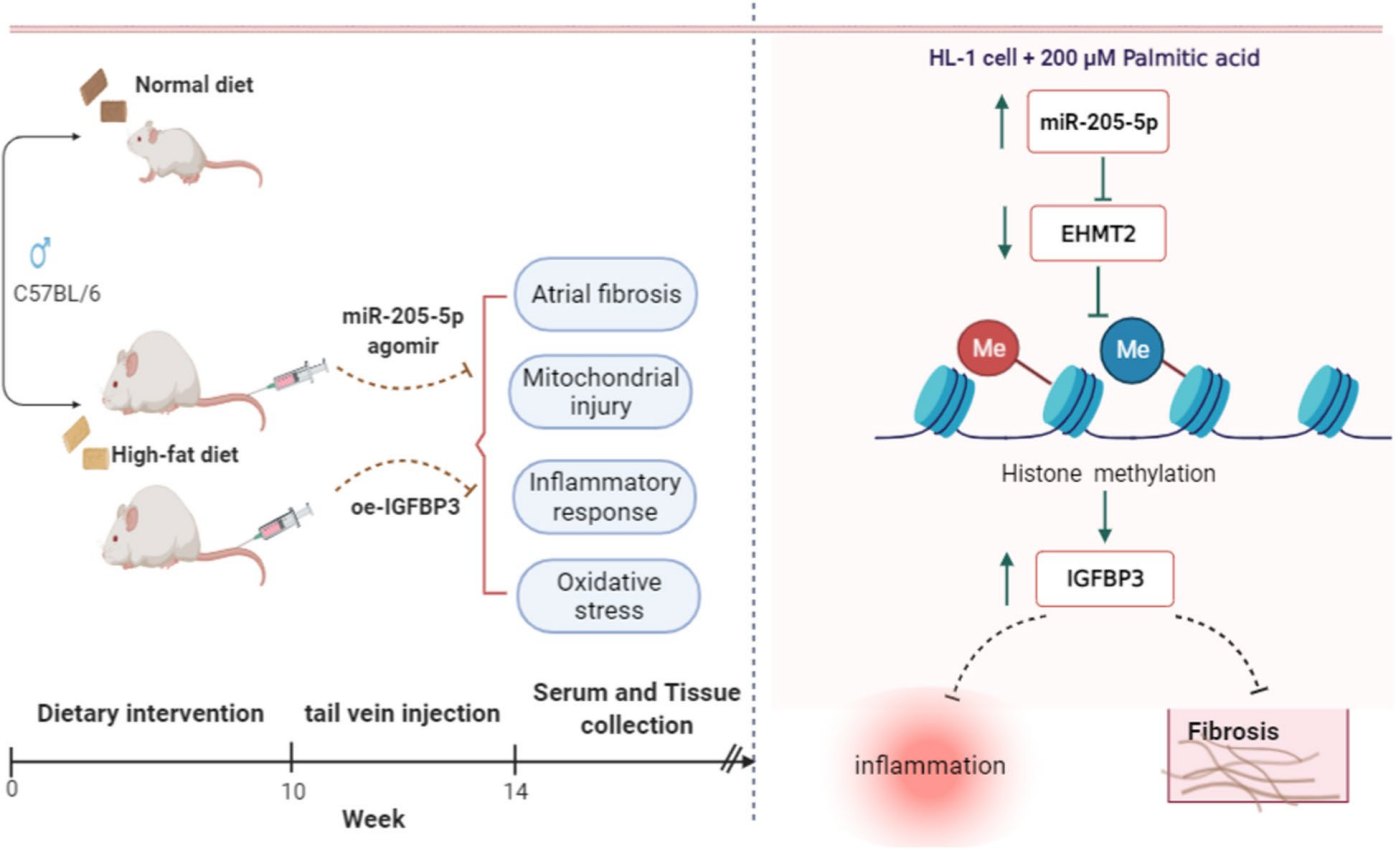

## Introduction

Atrial fibrillation (AF) is a cardiac arrhythmia typically manifested with palpitations, anhelation, and intolerance to exercise, relating to high morbidity, mortality, and medical costs [1]. AF can be induced by complex mechanisms involving diverse pathophysiological events in the atria, which poses a therapeutic challenge for clinicians [2]. It is widely recognized that the pathophysiological processes of AF encompass structural remodeling (wherein atrial fibrosis is a hallmark), electrical remodeling, and contractile remodeling of the atria [3]. Atrial fibrosis is a common characteristic of the structural remodeling owing to multifarious risk factors including heart failure, hypertension, and obesity, and also a pathophysiological contributor to the progression of AF related to relapse, therapy resistance, and complicating diseases [4, 5]. Reports have shown that obesity is an independent risk factor for AF [6, 7]. The degree of obesity is positively correlated with the risk of AF [8]. High fat-diet (HFD) mouse models have been used to study obesity-related AF in previous studies [9, 10]. Therefore, it is also of significance to explore the mechanism of obesity-induced atrial fibrosis in AF, for which a HFD mouse model can be used.

Complicated neurohumoral, cellular, and molecular interactions are implicated in atrial fibrosis, and an in-depth understanding about these mechanisms in the context of AF shows a remarkable significance for seeking therapeutic regimens targeting the fibrous region [11]. Additionally, mitochondrial dysfunction that elicits oxidative stress and calcium overload and inflammation in the atrial electrical remodeling, calcium handling, and structural remodeling are two important processes in the development of AF [12, 13]. Recent animal experiments and clinical trials have indicated the dysregulation of microRNA (miRNA) expression in atrial tissue and circulating blood and their impacts on AF-related structural and electrical remodeling, providing useful diagnostic biomarkers and therapeutic targets for this disease [14, 15].

MiRNAs are a battery of post-transcriptional mediators of the genes implicated in the pathophysiological processes of AF (such as cardiac automaticity and fibrosis) [16]. Investigations on these miRNAs shed light on the molecular mechanisms in the etiopathogenesis of AF and suggest miRNA targeting to be an appealing strategy for the treatment and management of this disorder [17]. Among these miRNAs, miR-205 has been recently illustrated to affect the progression of AF by regulating the functions of rat fibroblasts [18]. Meanwhile, miR-205-5p can act as an inhibitor of oxidative stress and inflammation in hypoxia/reoxygenation-injured rat hippocampal neurons [19].

EHMT2, also named G9a, is a euchromatic H3K9 methyltransferase that participates in the process of renal fibrosis through H3K9 modification [20]. Apart from H3K9 methylation, EHMT2 can also regulate H3K9 acetylation and H3K4 methylation [21]. It is reported that EHMT2 orchestrates critical epigenetic changes required for cardiomyocyte homeostasis and also regulates hypertrophy via H3K9me2 [22, 23]. Moreover, EHMT2 can mediate HFD-induced obesity, hepatic steatosis, and hepatic insulin resistance [24, 25], emerging as a therapeutic candidate for obesity and associated diseases. Hence, we sought to testify if there is a relationship between miR-205-5p and EHMT2 in the pathophysiological processes of obesity-induced atrial fibrosis and to investigate whether EHMT2-induced H3K9 or H3K4 modification underlies the dysregulated expression of genes related to AF.

## Materials and methods

### Ethics statement

This study was ratified by the Ethics Committee of Nanfang Hospital. The informed consent of each patient participating in the study was obtained. This study was implemented in accordance with the Declaration of Helsinki. All animal experiments complied with the rules and regulations of experimental animal management and operating standards as well as the ethical requirements of experimental animals. Additionally, all animal procedures were performed referring to the standards of humane animal care approved by the Medical Ethics Committee of Nanfang Hospital.

### Clinical samples

Forty patients with valvular heart disease who received thoracotomy valve replacement surgery in Nanfang Hospital from July 2017 to October 2020 were included in the study. According to the results of 12-lead electrocardiogram before surgery, patients were divided into a sinus rhythm (SR) group (*n* = 14, no history of AF, the basic heart rhythm was SR by electrocardiogram at admission, electrocardiograph monitoring and dynamic electrocardiogram, and no AF was indicated) and an AF group (*n* = 26). The left atrial appendicular tissues approximately 0.5 × 0.5 × 0.3 cm in size of all patients were obtained during cardiac surgery.

### Experimental animals

A total of 36 male C57BL/6 mice (aged 6–8 weeks old, and weighing 19–22 g) were available from GemPharmatech (Nanjing, China). Mice were raised on normal diet for 1 week in a specific pathogen free grade animal rearing room, maintained at a room temperature of 21–24 °C, a humidity of 50–60%, with a 12/12-h light/dark cycle.

### HFD-induced atrial fibrosis model in mice and grouping

C57BL/6 male mice were induced by HFD for the establishment of atrial fibrosis model [26, 27]. The 36 mice were randomly assigned into sham group, model group, agomir-negative control (NC) group, miR-205-5p agomir group, overexpression (oe)-NC group, and oe-IGFBP3 group (*n* = 6 per group). The sham group was fed with a standard diet for 10 weeks and the model group was fed with 60% kcal high-fat for 10 weeks, during which the mice were weighed at 1, 3, 5, 7, and 9 weeks. After 10 weeks of HFD treatment, the model group continued to receive no additional treatment except for HFD, while the agomir-NC, miR-205-5p agomir, oe-NC and oe-IGFBP3 groups were injected with agomir-NC, miR-205-5p agomir, oe-NC and oe-IGFBP3, respectively, for consecutive 4 weeks (twice a week), during which HFD was still provided. In these 4 weeks, the sham group continued to receive the standard diet. After these 14 weeks, the mice were anesthetized with 3% pentobarbital sodium (30 mg/kg) and euthanized, and left atrial tissues were harvested for hematoxylin–eosin (H&E) staining, Masson staining, and other factor detection.

miR-205-5p agomir and agomir-NC (agonist and control of miR-205-5p, GenePharma, Shanghai, China) were injected into mice via the tail vein at the dose of 30 pmol/g, and oe-IGFBP3 and oe-NC (lentiviral vector for overexpression of IGFBP3 and its control, 50 μL, GenePharma) were injected into mice via the tail vein with a virus titer of 1 × 10^8^ TU/mL.

### Echocardiogram

In order to evaluate the left atrium remodeling, the skin from the mouse chest and upper abdomen was prepared for echocardiogram after 10 weeks of HFD. Next, the mice were fixed on the operating table with a supine position and anesthetized with 3% pentobarbital sodium (30 mg/kg) to obtain an echocardiogram image to assess heart rate (HR), left atrial diameter (LAD), left ventricular end diastolic dimension (LVDd), and left ventricular posterior wall (LVPW). These indexes were assessed based on the methods of a previous study [28].

### Tissue treatment

The mice were euthanized, a median sternotomy was performed and the heart was quickly obtained. The heart was perfused with PBS, and then the heart tissues were cut after perfusion. The excess water was removed with filter papers, the vessels were washed, and the left atrial tissues were harvested after complete removal of the aorta and fat. One part of the left atrial tissues were taken for slice-making, placed in a centrifuge tube and fixed with 4% neutral formaldehyde solution; another part of tissues was used for separating the mitochondria, and the tissues were quickly cut and minced in ice-cold isolation medium (sucrose 17.115 g, HEPES 0.143 g, EDTA Na 20.037 g, distilled water 200 mL, pH 7.4). The remaining left atrial tissues were quickly cooled by liquid nitrogen and stored at – 80 °C for protein analysis or for further use. A previous study was referred to for the tissue collection [29].

### Isolation, treatment, and functional quantification of the mitochondria

Mitochondria were isolated as previously described [29]. The minced blood-free atrial tissues were homogenized six times (0–4 °C) using a manual glass homogenizer. Subsequently, the homogenates were centrifuged at 1000 × *g* for 10 min to collect the supernatant, followed by centrifugation at 10,000 × *g* for 10 min. The main component of the sediment was mitochondrial precipitation, which was suspended in 0.5 mL of medium (HEPES 0.143 g, potassium chloride 1.928 g, EDTA Na2 0.037 g, KH2PO4 0.054 g, bovine serum albumin 0.2 g, distilled water 200 mL, pH 7.4). The mitochondrial isolation was best completed within 1 h after euthanasia. Mitochondrial protein content was determined using the bicinchoninic acid (BCA) protein quantification kit (TIANGEN, Beijing, China). Western blot analysis was implemented to assess the expression levels of mitochondrial biogenesis-, division-, and fusion-related proteins in the left atrial tissues.

### Transmission electron microscope (TEM) observation

The mitochondrial superstructure in the left atrial tissues was visualized by a TEM (HT-7700, Hitachi, Tokyo, Japan). Samples were fixed with 2.5% glutaraldehyde, rinsed with PBS, and fixed with 1% osmium tetroxide for 1 h. Samples were dehydrated in graded ethanol and acetone, embedded in epoxy resin and sectioned to 70 nm. Next, the sections were placed on a TEM grid and stained, and finally amplified for observation and imaging under a TEM.

### H&E staining and Masson staining

Before staining, the paraffin-embedded atrial tissue Sects. (3–5 μm) were dried in a 65 °C incubator for 1 h. H&E staining and Masson staining were performed according to standard protocols. The slides were visualized by an optical microscopy and photographed to assess histopathology and the degree of fibrosis, respectively.

### Determination of serum blood lipid levels

The serum levels of total cholesterol (TC; A111-1–1), triglycerides (TG; A110-1–1), low-density lipoprotein cholesterol (LDL-C; A113-1–1), and high-density lipoprotein cholesterol (HDL-C; A112-1–1) were measured using the kits (Nanjing Jiancheng Bioengineering Institute, Nanjing, China), and the experimental operations were conducted as per the instructions of the corresponding kits and the methods of a previous study [29].

### Terminal deoxynucleotidyl transferase-mediated nick end labeling (TUNEL) staining

Tissue apoptosis was detected using a TUNEL kit (Roche, Mannheim, Germany) in strict accordance with the kit instruction and the documented methods [30]. In brief, the samples were fixed, washed in PBS and then incubated with 50 μL TUNEL reaction mixture (5 μL TdT + 45 μL fluorescein labeled dUTP solution) at 37 °C for 60 min. Finally, the tissues were captured by using a fluorescence microscope. Apoptotic cells and total cells were counted with the Image Pro Plus 6.0 image analysis software, and the percentage of apoptotic cells in the number of total cells was calculated, this was, the apoptotic index.

### Evaluation of oxidative stress

To evaluate oxidative stress, the contents of oxidative damage indicators malondialdehyde (MDA; A003-1–2) and superoxide dismutase (SOD; A001-1–2) in the left atrial tissues were determined according to the kit instructions (Nanjing Jiancheng Bioengineering Institute).

### Cell culture and treatment

Mouse atrial muscle cells (HL-1, the American Type Culture Collection, ATCC, Manassas, VA, USA) were cultured in Claycomb medium (Sigma-Aldrich, St. Louis, MO, USA). Forty-eight hours before the experiment, 5 × 10^5^ cells were seeded onto 6-well plates. HL-1 cells were treated with 200 μM palmitate acid (PA) for 24 h to construct a cell model of atrial muscle fibrosis [26].

### Cell transfection and grouping

MiR-205-5p-mimic, EHMT2 interference (sh-EHMT2), overexpression (oe-EHMT2) lentiviral vector, interference lentiviral vector of IGFBP3 (sh-IGFBP3), and their controls (miR-NC, sh-NC, and oe-NC) were purchased from GenePharma (Shanghai, China). Transfection of cells was carried out using Lipofectamine 2000 reagent (Invitrogen, Carlsbad, CA, USA) based on the instructions. The concentration of miR-205-5p-mimics transfection was 50 nM. Virus titers were determined using the p24 ELISA kit (Cell Biolabs, Inc., San Diego, USA). The virus titer of 5 × 10^8^ TU/mL was used in this study, and polybrene at a final concentration of 8 g/mL was added.

Cells were separated into the following groups: control group (normal control group, without PA treatment), PA group (model group, PA treatment for inducing fibrosis of atrial muscle cells), blank group (blank control group, with PA treatment and no transfection), mimic-NC group (PA + mimic-NC transfection), miR-205-5p-mimic group (PA + miR-205-5p-mimic transfection), sh-NC group (PA + sh-NC transfection), sh-EHMT2 group (PA + sh-EHMT2 transfection), miR-205-5p-mimic + oe-NC group (PA + transfections of miR-205-5p-mimic and oe-NC), miR-205-5p-mimic + oe-EHMT2 group (PA + transfections of miR-205-5p-mimic and oe-EHMT2), miR-205-5p-mimic + sh-NC group (PA + transfections of miR-205-5p-mimic and sh-NC), and miR-205-5p-mimic + sh-IGFBP3 group (PA + transfections of miR-205-5p-mimic and sh-IGFBP3).

### Reverse transcription quantitative polymerase chain reaction (RT-qPCR)

Total RNA was extracted with TRIZOL (Invitrogen, Carlsbad, CA, USA). Reverse transcription was implemented using a reverse transcription kit (TaKaRa, Tokyo, Japan), and all operations were performed based on the instructions of the kit. Gene expression was detected according to the documented methods [26] using a LightCycler 480 (Roche Diagnostics, Indianapolis, IN, USA), and reaction conditions were conducted referring to the operating instructions of the qPCR kit (SYBR Green Mix, Roche Diagnostics). The qPCR was set in three replicates per reaction. U6 was used as the internal reference of miRNA and glyceraldehyde phosphate dehydrogenase (GAPDH) as the internal reference of mRNA. Data analysis was processed using the 2^−ΔΔCt^ method. The amplification primer sequences of each gene and its internal reference are detailed in Table 1.

**Table 1:** Primer sequences for RT-qPCR

Gene	Sequence (5′-3′)
miR-205-5p-F	TCCTTCATTCCACCGGAGTCTG
miR-205-5p-R	GCGAGCACAGAATTAATACGAC
EHMT2-F	GCCAGGCCGGGAGGCCCTGGAA
EHMT2-R	CTCCAGCCTGCAGCAGCACATG
IGFBP3-F	GGTGTCTGATCCCAAGTTCC
IGFBP3-R	ACCATATTCTGTCTCCCGCT
TGF-β1-F	AACTCCGGTGACATCAAAAGATAA
TGF-β1-R	TGCTGAGGCTCAAGTTAAAAGT
U6-F	CTCGCTTCGGCAGCACA
U6-R	ACGCTTCACGAATTTGCGT
GAPDH-F	CCCTTAAGAGGGATGCTGCC
GAPDH-R	ACTGTGCCGTTGAATTTGCC

*F* forward, *R* reverse, *miR* microRNA, *GAPDH* glyceraldehyde phosphate dehydrogenase

### Western blot analysis

Protein samples were obtained by lysing the mouse left atrial tissues or atrial muscle cells using radioimmunoprecipitation assay (RIPA) lysis buffer (Beyotime, Shanghai, China) and the protein concentration was measured with a BCA kit (Beyotime) [31]. Next, a corresponding volume of protein was added with the loading buffer (Beyotime), mixed, and heated in boiling water bath for 3 min for denaturation. Subsequently, the proteins were separated with sodium dodecyl sulfate polyacrylamide gel electrophoresis and then electroblotted onto polyvinylidene fluoride membrane. Next, the membrane was rinsed in the wash solution for 1–2 min, and then blocked in the closed solution for 60 min at room temperature. After that, the membrane was cultured with the primary antibody against EHMT2 (ab194286, 1:1000), IGFBP3 (ab193910, 1:1000), TIMP1 (ab211926, 1:1000), α-SMA (ab5694, 1:1000), GATA4 (ab256782, 1:1000), COL I (ab34710, 1:1000), COL III (ab184993, 1:1000), PGC-1α (ab188102, 1:1000), NRF-1 (ab34682, 1:1000), Tfam (CST, #7495, 1:1000), DRP-1 (ab184247, 1:1000), Mfn-1 (ab129154, 1:1000), cleaved caspase-3 (ab32042, 1:1000), TGF-β (ab215715, 1:1000), Nav1.5 (CST, #14,421, 1:1000), CaV1.2 (ab84814, 1:500), KV1.5 (ab110469, 1:500), and GAPDH (ab9485, 1:1000) (all from Abcam [Cambridge, UK] except for Nav1.5 and Tfam), followed by 3-time washing, 10 min each time. Next, the membrane was incubated for 1 h with horseradish peroxidase-labeled goat anti-rabbit IgG (1:5000, Beijing ComWin Biotech Co., Ltd., Beijing, China) or rabbit anti-mouse IgG (ab6278, 1:500, Abcam), followed by 3-time washing, 10 min each time. Lastly, the membrane was dropped with developer, and the detection was performed using a chemiluminescence imaging system (Bio-Rad Laboratories, Hercules, CA, USA).

### Enzyme-linked immunosorbent assay (ELISA)

The concentrations of hs-CRP (E-EL-M0677c, R&D, USA), TNF-α (PT512, Beyotime), and IL-1β (PI301, Beyotime) in each group of cells or mouse left atrium tissues were measured using the ELISA kits, and all operations were implemented in strict accordance with the protocols of the corresponding kits and the literature [32].

### Chromatin immunoprecipitation (ChIP) assay

The cells were treated with 4% formaldehyde (final concentration of 1%), sonicated, and added with H3K4me3 antibody (Abcam, ab8580, use 2 μg for 25 μg of chromatin) or H3K9me2 antibody (Abcam, ab176882, use 2 μg for 25 μg of chromatin) for the binding with TRIM4 promoter region. Next, Protein A Agarose/SaLmon Sperm DNA was added for binding the gene promoter complex. The precipitated complex was washed to remove some non-specific binding. After elution, the enriched TRIM4 gene promoter complex was obtained and then decrosslinked. The enriched TRIM4 gene promoter fragment was purified for qPCR. The ChIP experiments were performed as previously described [33, 34].

### Dual-luciferase reporter gene assay

As per the results predicted by the starBase database, the wild and mutant sequences (WT-EHMT2 and MUT-EHMT2) of the binding site between miR-205-5p and EHMT2 were designed and synthesized. Dual-luciferase reporter gene assays were performed to verify the binding of miR-205-5p and EHMT2 [35, 36]. The wild and mutant sequences of the binding site were inserted into the pMIR-reporter reporter plasmid, and then co-transfected into HEK293T cells with miR-205-5p-mimic (30 nM) or its NC (30 nM), respectively. Cells were harvested and lysed 48 h after transfection, and then centrifuged for 3–5 min to collect the supernatant. The luciferase activity in the cell extracts was analyzed with a luciferase assay kit (Dual-Luciferase Reporter Assay System, Promega, USA). The ratio of the target luminescence to the reference luminescence was used as the relative luciferase activity and the luciferase intensities were measured by using a fluorescence detector (Promega).

### RNA immunoprecipitation (RIP) assay

RIP was adopted for verifying the binding of miR-205-5p and EHMT2 [35, 36], and a RIP kit (17–701, Millipore, USA) was used here. In short, the cells were taken, and the supernatant was removed after washing with pre-cooled PBS. Cells were lysed with an equal volume of RIPA lysis buffer (P0013B, Beyotime) and ice-bathed for 5 min, and the supernatant was removed at 14,000 rpm by centrifugation at 4 °C for 10 min. Part of the cell extract was isolated as input and another part was cultured with antibodies for co-precipitation. The specific step was: 50 μL of magnetic beads were washed for each co-precipitation reaction system and resuspended with 100 μL of RIP Wash Buffer, and 5 μg of antibody was added according to the experimental grouping. The magnetic bead-antibody complex was rinsed and then resuspended with 900 μL of RIP Wash Buffer, followed by incubation overnight with 100 μL of cell extract at 4 °C. Samples were rinsed 3 times and then put on magnetic rack to collect the magnetic beads-protein complex. Antibody used for RIP was the rabbit anti-Ago2 (1:50, ab186733, Abcam), which was mixed at room temperature for 30 min, and the rabbit anti-IgG (1:100, ab172730, Abcam) was utilized as the NC.

### Statistical analysis

Statistical analysis was performed with GraphPad Prism 6 software, and all data were represented as mean ± standard deviation. Comparisons between two groups were performed by the t test, and comparisons among multiple groups were performed using one-way analysis of variance test, followed by Tukey’s multiple comparisons test. *P* less than 0.05 were considered as statistically significant difference.

## Results

### Decreased miR-205-5p expression and increased EHMT2 expression in AF patients

Evidence has shown that miR-205 is poorly expressed in angiotensin II-induced rat atrial fibrosis models [18]. Meanwhile, there were significant increases in EHMT2 expression and H3K9 methylation levels in TGF-β1-induced renal fibrosis mice and bleomycin-induced pulmonary fibrosis mice [20, 37]. However, there was currently no study of EHMT2 in atrial fibrosis.

**Fig. 1:**
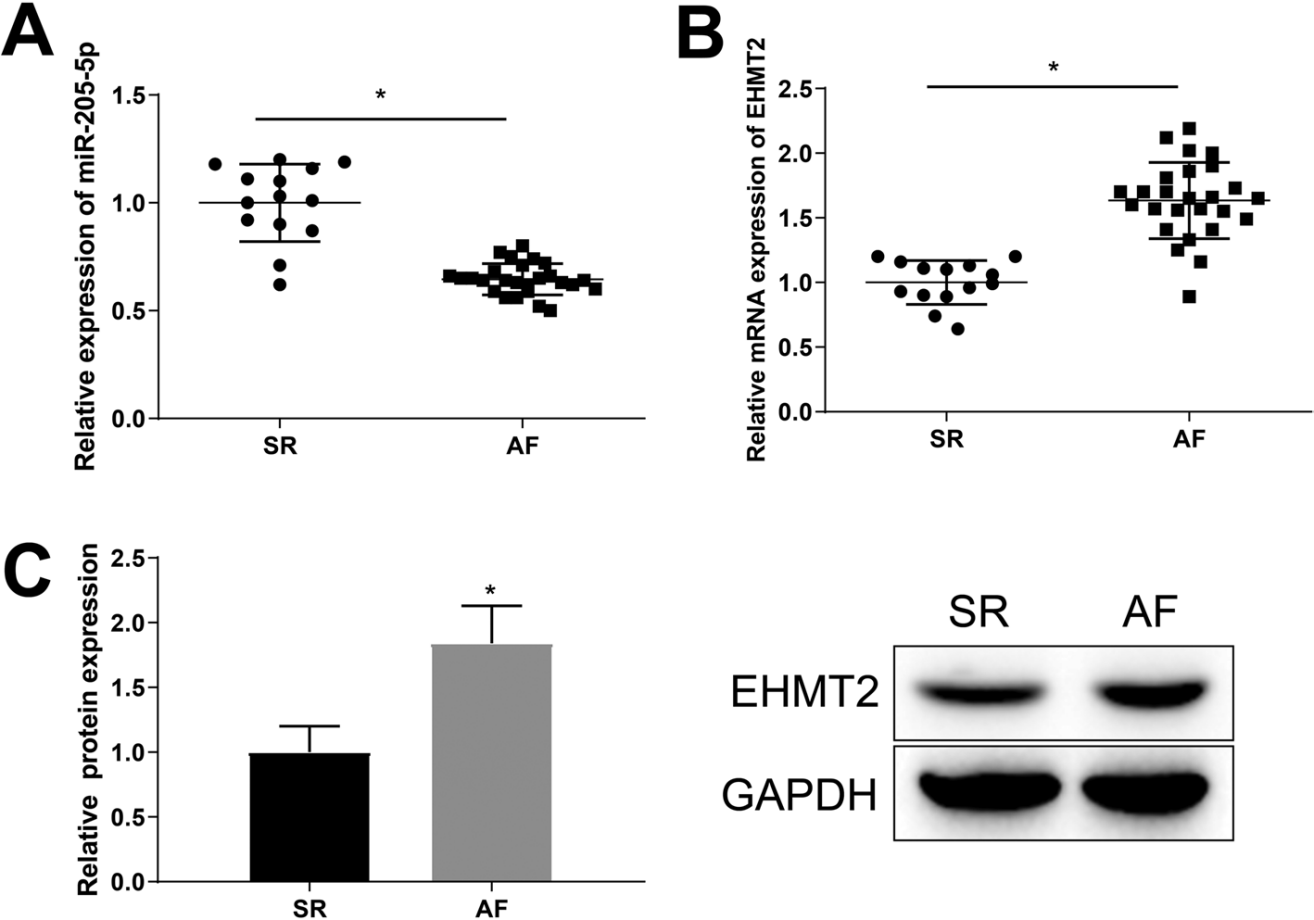
miR-205-5p is lowly expressed and EHMT2 is highly expressed in AF patients. **A** The expression of miR-205-5p in serum was tested by RT-qPCR. **B**, **C**. The expression of EHMT2 in serum was tested by RT-qPCR and western blot analysis. *p < 0.05 vs. the SR group

Forty patients were assigned into the AF group (26 cases) and the SR group (14 cases), and no significant difference was observed in the age, gender and ejection fraction (EF) value between the two groups (all *p* > 0.05), and LAD in the AF group was longer than that in the SR group (*p* < 0.05) (Table 2). The expression of miR-205-5p and EHMT2 in the atrial appendicular tissues of AF patients was tested by RT-qPCR and western blot analysis, which showed that miR-205-5p expression was lowered 0.64-fold and EHMT2 expression was elevated (mRNA: 1.63-fold; protein: 1.84-fold) in the AF group in comparison to the SR group (Fig. 1A–C).

**Table 2:** Comparison of the general clinical data between the AF group and SR group

Clinicopathological factors	AF group (26 cases)	SR group (14 cases)	*p*
Gender			
Male	11	8	> 0.99
Female	15	6	
Age (years)			
≤ 42	13	6	> 0.99
> 42	13	8	
Echocardiographic parameters			
LAD (mm)	59.25 ± 11.36	43.84 ± 11.54	0.0002*
LVDd (mm)	53.43 ± 8.45	52.48 ± 7.68	> 0.99
LVDs (mm)	35.16 ± 1.27	34.53 ± 1.32	> 0.99
EF (mm)	53 ± 2.1	52 ± 2.4	> 0.99

*LAD* left atrium diameter, *LVDd* left ventricular dimension in diastole, *LVDs* left ventricular dimension in systole, *EF* ejection fraction

*p < 0.05

### Upregulation of miR-205-5p attenuates fibrosis of atrial muscle cells

**Fig. 2:**
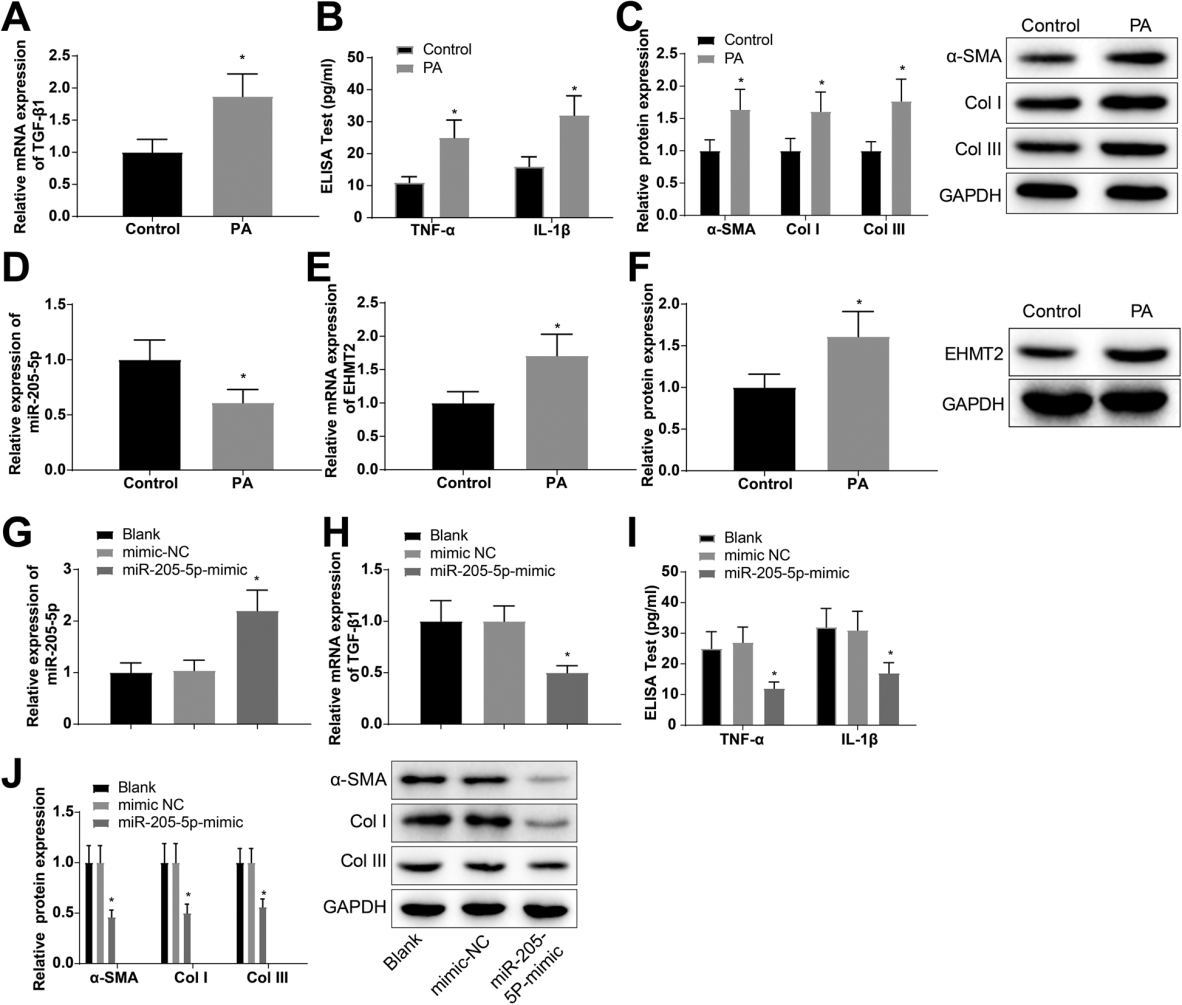
Overexpression of miR-205-5p attenuates fibrosis in atrial muscle cells. HL-1 cells were with 200 μM PA for 24 h for establishing a cell model of atrial fibrosis (recorded as the PA group). **A** TGF-β1 expression in cells was determined by RT-qPCR. **B** The secretion of inflammatory factors TNF-α and IL-1β in cells was measured by ELISA. **C** Expression of fibrosis-related proteins α-SMA, Col I and COL III in cells was determined by western blot analysis. **D**–**F**. miR-205-5p and EHMT2 levels in cells were determined by RT-qPCR and western blot analysis. Transfection of miR-205-5p-mimic and mimic-NC in HL-1 cells. **G** Expression of miR-205-5p in the transfected cells was determined using RT-qPCR. H. TGF-β1 expression in transfected cells was determined by RT-qPCR. I. The secretion of cell inflammatory factors TNF-α and IL-1β in transfected cells was measured by ELISA. **J** Expression of fibrosis-related proteins α-SMA, Col I, and COL III in transfected cells was determined by western blot analysis. *p < 0.05, vs. the control group or the mimic-NC group

To explore the effect of miR-205-5p on atrial fibrosis at the cellular level, we treated HL-1 cells with 200 μM PA for 24 h and constructed a cell model of atrial fibrosis (recorded as the PA group). TGF-β1 expression in cells was tested by RT-qPCR, the secretion of cell inflammatory factors TNF-α and IL-1β by ELISA, and the expression of fibrosis-related proteins α-smooth muscle actin (α-SMA), collagen I (Col I), and collagen III (COL III) by western blot analysis. The results indicated that the expression levels of TGF-β1 (1.87-fold), TNF-α (2.27-fold), and IL-1β (2.0-fold) were increased in the PA group versus the control group, and those of α-SMA (1.64-fold), Col I (1.61-fold), and COL III (1.77-fold) were also elevated, indicating the successful construction of the cell model of atrial fibrosis stimulated by PA (Fig. 2A–C). Meanwhile, miR-205-5p and EHMT2 expression in cells was examined by RT-qPCR and western blot analysis, which suggested that cells in the PA group exhibited reduced miR-205-5p expression (0.61-fold) and increased EHMT2 expression (mRNA: 1.71-fold; protein: 1.61-fold) versus the control group (Fig. 2D-F).

After transfection of miR-205-5p-mimic and mimic-NC in HL-1 cells, the expression of miR-205-5p in the transfected cells was determined using RT-qPCR. The findings revealed that cells transfected with miR-205-5p-mimic showed a 2.11-fold elevation of miR-205-5p expression (Fig. 2G), indicating the effectiveness of miR-205-5p-mimic transfection. Additionally, there were reductions in the expression levels of TGF-β1 (0.5-fold), TNF-α (0.44-fold), IL-1β (0.55-fold), α-SMA (0.46-fold), Col I (0.50-fold), and COL III (0.56-fold) in cells upon miR-205-5p-mimic transfection (Fig. 2H-J). It is suggested that overexpression of miR-205-5p attenuates fibrosis of atrial muscle cells.

### miR-205-5p modulates the fibrosis of atrial muscle cells through binding to EHMT2

**Fig. 3:**
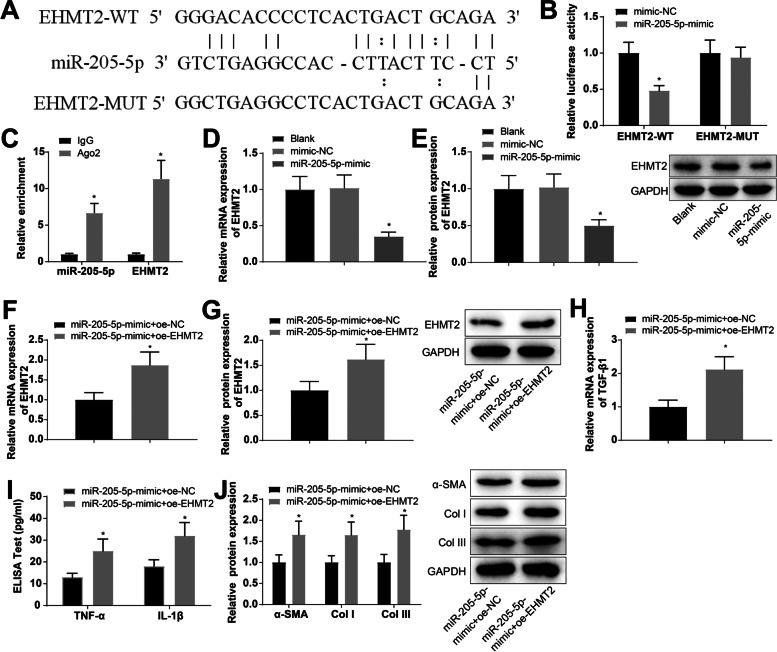
miR-205-5p targets EHMT2. **A** The Starbase database predicted the binding site of miR-205-5p and EHMT2. **B**, **C**. Luciferase activity and RIP assays were carried out to detect the binding relationship of miR-205-5p and EHMT2. D-E. EHMT2 expression in cells transfected with miR-205-5p-mimic was determined by RT-qPCR and western blot analysis. **F**, **G** Expression of EHMT2 in cells co-transfected with miR-205-5p-mimic and oe-EHMT2 was determined using RT-qPCR and western blot. **H** TGF-β1 expression in cells co-transfected with miR-205-5p-mimic and oe-EHMT2 was determined by RT-qPCR. **I** The secretion of cell inflammatory factors TNF-α and IL-1β in cells co-transfected with miR-205-5p-mimic and oe-EHMT2 was measured by ELISA. **J** Expression of fibrosis-related proteins α-SMA, Col I, and COL III in cells co-transfected with miR-205-5p-mimic and oe-EHMT2 was determined by western blot analysis. *p < 0.05, vs. the IgG group, the mimic-NC group or the miR-205-5p-mimic + oe-NC group

The starBase database predicted that miR-205-5p had binding sites for EHMT2 (Fig. 3A), and the association of miR-205-5p and EHMT2 was detected using a luciferase activity assay. The results indicated that the co-transfection of miR-205-5p-mimic and EHMT2-WT reduced the luciferase activity 0.48-fold (Fig. 3B). According to RIP assay, miR-205-5p and EHMT2 were increased 6.65-fold and 11.32-fold by Ago2 (Fig. 3C). The expression of EHMT2 in the miR-205-5p-overexpressing cells was determined using RT-qPCR and western blot analysis. The results revealed that cells transfected with miR-205-5p-mimic showed reductions in EHMT2 mRNA and protein expression (0.34-fold and 0.49-fold, respectively) (Fig. 3D, E).

In HL-1 cells which were transfected with miR-205p-mimic + oe-EHMT2 and miR-205-5p-mimic + oe-NC, EHMT2 expression was detected using RT-qPCR and western blot analysis. The results showed that oe-EHMT2 promoted EHMT2 expression (mRNA: 1.87-fold; protein: 1.62-fold) despite of miR-205-5p mimic transfection (Fig. 3F, G). Meanwhile, compared with miR-205-5p-mimic transfection alone, oe-EHMT2 transfection in the presence of miR-205-5p-mimic increased the expression levels of TGF-β1 (2.12-fold), TNF-α (1.92-fold), IL-1β (1.77-fold), α-SMA (1.66-fold), Col I (1.65-fold), and COL III (1.78-fold) (Fig. 3H–J). The results indicate that miR-205-5p affects fibrosis of atrial muscle cells through the negative regulation of EHMT2 expression.

### miR-205-5p elevates IGFBP3 expression by regulating the methylation of H3 histones by binding to EHMT2

**Fig. 4:**
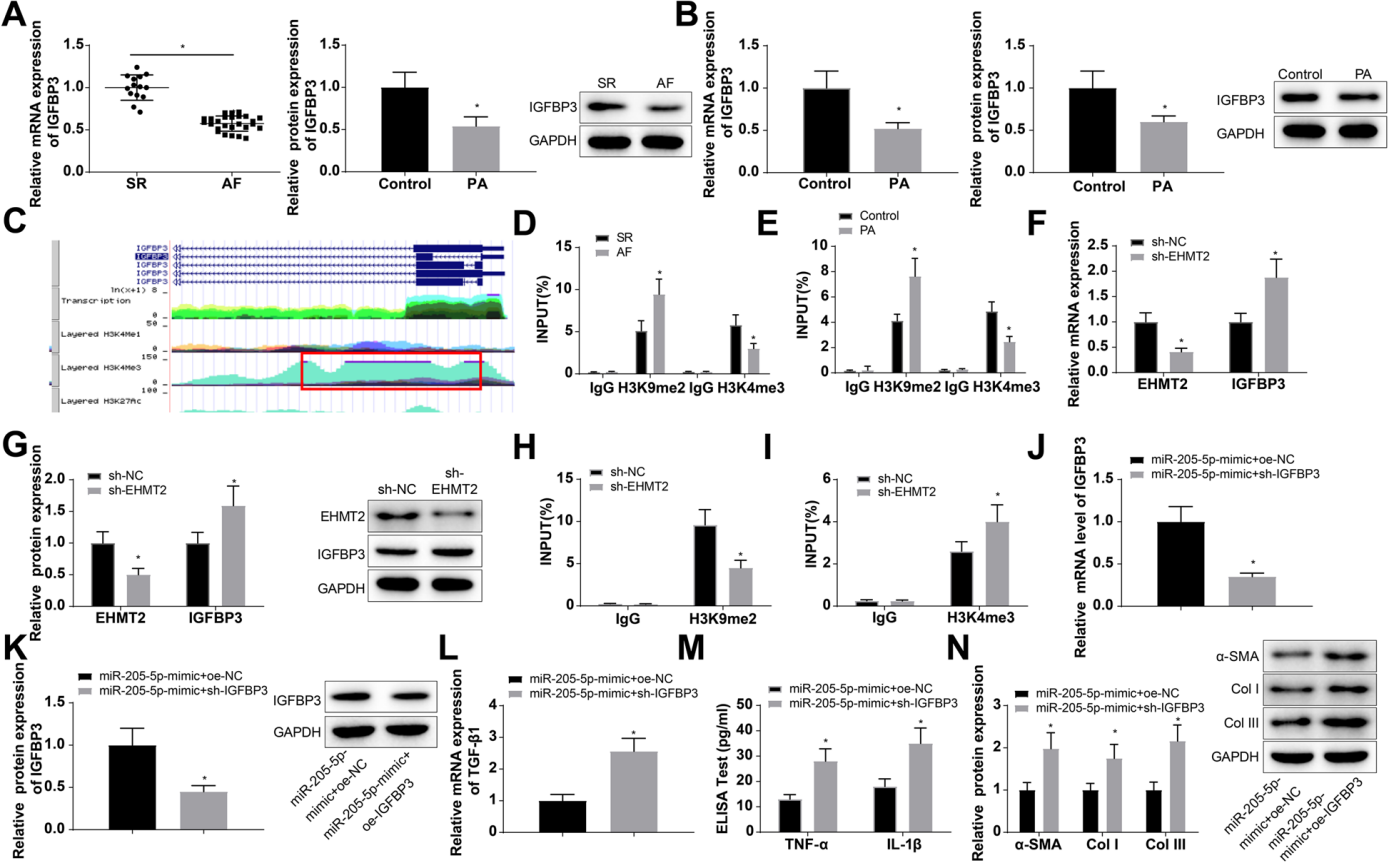
miR-205-5p elevates IGFBP3 expression by regulating the methylation of H3 histones by binding to EHMT2. **A**, **B** The expression of IGFBP3 in clinical tissues or PA-treated fibrotic cells using RT-qPCR and western blot analysis. **C** Prediction analysis of the UCSC database. **D**, **E** ChIP assay was implemented to detect the H3K9me2 and H3K4me3 enrichment in IGFBP3 promoter region in clinical tissues and cell models. **F**, **G**. EHMT2 and IGFBP3 expression levels in cells transfected with sh-EHMT2 were tested by RT-qPCR and western blot analysis. **H**, **I** H3K9me2 enrichment and H3K4me3 enrichment in IGFBP3 promoter region in cells transfected with sh-EHMT2 were assessed by ChIP assay. **J**, **K** Expression of IGFBP3 in cells co-transfected with miR-205-5p-mimic and sh-IGFBP3 was determined using RT-qPCR and western blot analysis. **L** TGF-β1 expression in cells co-transfected with miR-205-5p-mimic and sh-IGFBP3 was determined by RT-qPCR. **M** The secretion of cell inflammatory factors TNF-α and IL-1β in cells co-transfected with miR-205-5p-mimic and sh-IGFBP3 was measured by ELISA. **N** Expression of fibrosis-related proteins α-SMA, Col I and COL III in cells co-transfected with miR-205-5p-mimic and sh-IGFBP3 was determined by western blot analysis. *p < 0.05, vs. the SR group, the control group, the sh-NC group or the miR-205-5p-mimic + sh-NC group

IGFBP3 is a member of the IGFBPs, and it has been revealed that low serum levels of IGFBP3 and IGF-1 are independently associated with AF [38]. The expression of IGFBP3 in clinical tissues or PA-treated fibrotic cells detected using RT-qPCR and western blot analysis showed that IGFBP3 expression was lowered (mRNA: 0.57-fold; protein: 0.54-fold) in the atrial appendicular tissues of AF patients (Fig. 4A). IGFBP3 expression was also reduced (mRNA: 0.52-fold; protein: 0.6-fold) in PA-treated fibrotic cells (Fig. 4B).

The UCSC database showed a peak of histone H3 methylation in the IGFBP3 promoter region (Fig. 4C), indicating that IGFBP3 was regulated by H3 methylation. Subsequently, ChIP assay was implemented to detect the H3K9me2 and H3K4me3 enrichment in IGFBP3 promoter region in clinical tissues and cell models, and the results revealed an increased H3K9me2 enrichment and a reduced H3K4me3 enrichment in AF patients, and the trend of ChIP results was the same in the cell model (Fig. 4D, E).

With the aim to explore the effect of EHMT2 on IGFBP3 expression and methylation levels, we examined EHMT2 and IGFBP3 expression in cells transfected with sh-EHMT2 by RT-qPCR and western blot analysis. The findings demonstrated a reduction in EHMT2 expression (mRNA: 0.41-fold; protein: 0.50-fold) and an increase in IGFBP3 expression (mRNA: 1.88-fold; protein: 1.59-fold) in cells transfected with sh-EHMT2 (Fig. 4F, G). Next, ChIP results for detecting IGFBP3 promoter region enrichment in transfected cells showed a decreased H3K9me2 enrichment and an increased H3K4me3 enrichment in IGFBP3 promoter region in cells transfected with sh-EHMT2 (Fig. 4H, I). The above-mentioned results indicate that EHMT2 inhibits IGFBP3 expression by modulating H3 histone methylation, which may be controlled by upregulating H3K9me2 or downregulating H3K4me3 methylation. There may be a functional competition between H3K4 and H3K9 methylation in atrial muscle cells.

To further explore whether miR-205-5p affected the fibrosis of atrial muscle cells by regulating IGFBP3 expression, HL-1 cells were transfected with miR-205-5p-mimic + sh-NC and miR-205-5p-mimic + sh-IGFBP3. IGFBP3 expression in the transfected cells was detected using RT-qPCR and western blot analysis, and the findings indicated that sh-IGFBP3 reduced IGFBP3 expression (mRNA: 0.35-fold; protein: 0.45-fold) in the presence of miR-205-5p-mimic (Fig. 4J, K). Meanwhile, compared with the miR-205-5p-mimic + sh-NC transfection, the miR-205-5p-mimic + sh-IGFBP3 transfection increased the expression of TGF-β1 (2.56-fold), TNF-α (2.15-fold), IL-1β (1.94-fold), α-SMA (1.98-fold), Col I (1.75-fold), and COL III (2.16-fold) (Fig. 4L–N). The findings implied that miR-205-5p overexpression induces IGFBP3 expression through regulating the methylation of H3 histones by binding to EHMT2, which subsequently inhibits the fibrosis of atrial muscle cells.

### Establishment of an HFD-induced atrial fibrosis model in mice

**Fig. 5:**
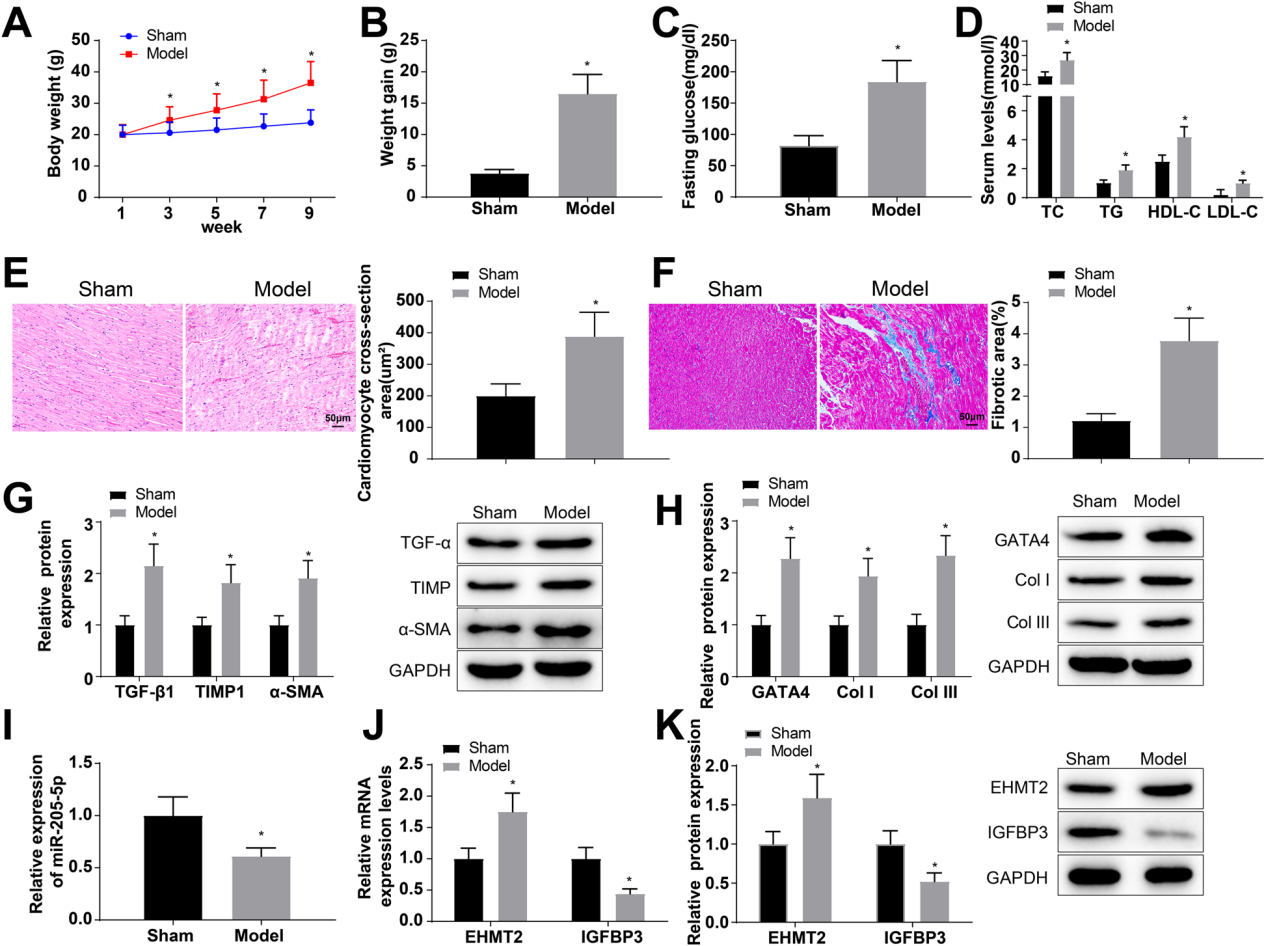
Establishment of a HFD-induced atrial fibrosis model in mice. **A** Weight changes in mice during the experiment. **B** Weight gain of mice at the end of the experiment. **C** Fasting blood glucose level of mice. **D** ELISA was utilized for measuring the serum levels of TC, TG, HDL-C, and LDL-C. **E** Pathology of the left atrial tissues in mice was observed by H&E staining. **F** Masson staining was conducted for observing collagen deposition and atrial fibrosis. **G**, **H** Western blot analysis was conducted to examine the protein expression of key regulatory proteins of fibrosis (TGF-β1, TIMP1, and α-SMA) and hypertrophic markers (GATA4, Col I, and COL III). **I**–**K** Expression of miR-205-5p, EHMT2 and IGFBP3 in the left atrial tissues of mice was determined using RT-qPCR and western blot analysis. *p < 0.05, vs. the sham group

Obesity or HFD causes susceptibility to atrial remodeling and atrial arrhythmias. In order to figure out the mechanism of miR-205-5p and IGFBP3 on HFD-induced atrial fibrosis at the animal level, we used HFD-induced mice to establish an atrial fibrosis model (named as a model group). Mouse weight increased throughout the experimental process, and mice in the model group were consistently heavier than those in the sham group. At 9th week of HFD feeding, weight, weight gain, and fasting glucose levels were higher in the model group than in the sham group (Fig. 5A–C). The measurement of TC, TG, HDL-C, and LDL-C by ELISA showed an increase in serum lipid levels in the model group versus the sham group (Fig. 5D), indicating that HFD induced obesity and caused dyslipidemia in mice. Next, the cardiac echocardiography assessments showed significantly lower HR and significantly higher LAD in model rats (Table 3), suggesting that an abnormal atrial function occurred in mice fed with a HFD.

**Table 3:** Echocardiographic analysis

Group	Sham	Model	*p* value
HR (bpm)	412 ± 41	367 ± 34	0.045*
LAD (mm)	2.1 ± 0.31	2.7 ± 0.33	0.0088*
LVDd (mm)	3.61 ± 0.61	3.95 ± 0.66	0.71
LVPW (mm)	0.78 ± 0.08	0.66 ± 0.07	0.27
LVDs (mm)	2.18 ± 0.21	2.22 ± 0.23	0.75
EF (%)	54 ± 6.5	51 ± 6.2	0.43

*HR* heart rate during the echocardiographic measurement, *LAD* left atrium diameter, *LVDd* left ventricular dimension in diastole, *LVPW* left ventricular posterior wall thickness during diastole, *LVDs* left ventricular dimension in systole, *EF* ejection fraction. *p < 0.05, vs. the sham group

After 14 weeks, the pathology of the left atrial tissues of in mice was observed using H&E staining. Mice in the sham group showed complete and regular atrial myocardial structure with less severe inflammasome infiltration, but mice in the model group presented disordered atrial muscle fibers with uneven orientation, significantly increased cross-sectional area of cardiomyocytes, and exacerbated inflammasome infiltration (Fig. 5E). The collagen deposition and atrial fibrosis were observed by Masson staining. It was observed that the ventricular collagen deposition was increased and the myocardial fibrosis was severe in mice of the model group versus the sham group (Fig. 5F). Western blot analysis was implemented to examine the protein expression of key regulatory proteins of fibrosis (TGF-β1, TIMP1, and α-SMA) and hypertrophic markers (GATA4, Col I, and COL III). The expression of TGF-β1, TIMP1, α-SMA, GATA4, Col I, and COL III was found to be upregulated 2.15-fold, 1.82-fold, 1.91-fold, 2.28-fold, 1.94-fold, and 2.34-fold, respectively, in mice of the model group in comparison to the sham group (Fig. 5G, H). The above results indicate that a HFD induces obese atrial fibrosis in mice. Next, the expression of miR-205-5p, EHMT2 and IGFBP3 in the left atrial tissues of mice was determined using RT-qPCR and western blot analysis. The results suggested decreases in the expression of miR-205-5p (0.61-fold) and IGFBP3 (mRNA: 0.44-fold; protein: 0.52-fold) and increases in EHMT2 mRNA and protein expression (1.75-fold and 1.59-fold, respectively) in mice of the model group when compared to the sham group (Fig. 5I–K). It is suggested that miR-205-5p, IGFBP3, and EHMT2 in mouse models might participate in the development of atrial fibrosis.

### Upregulation of miR-205-5p or IGFBP3 ameliorates the atrial fibrosis and mitochondrial damage caused by HFD

It has been shown that diabetic mice induced by HFD and streptozocin (STZ) injection have abnormal responses like dyslipidemia, oxidative stress, inflammatory response, atrial enlargement and fibrosis [39]. Long-term HFD has also been revealed to induce cardiac hypertrophy and atrial fibrosis in mice [40]. For the purpose of exploring the effect of miR-205-5p and IGFBP3 expression on HFD-induced atrial fibrosis, the model mice were randomly divided into agomir-NC, miR-205-5p agomir, oe-NC, and oe-IGFBP3 groups. Mice were respectively injected with agomir-NC, miR-205-5p agomir, oe-NC, and oe-IGFBP3 via the tail vein twice a week. They were euthanized after 4 weeks of injection and the left atrial tissues were harvested.

**Fig. 6:**
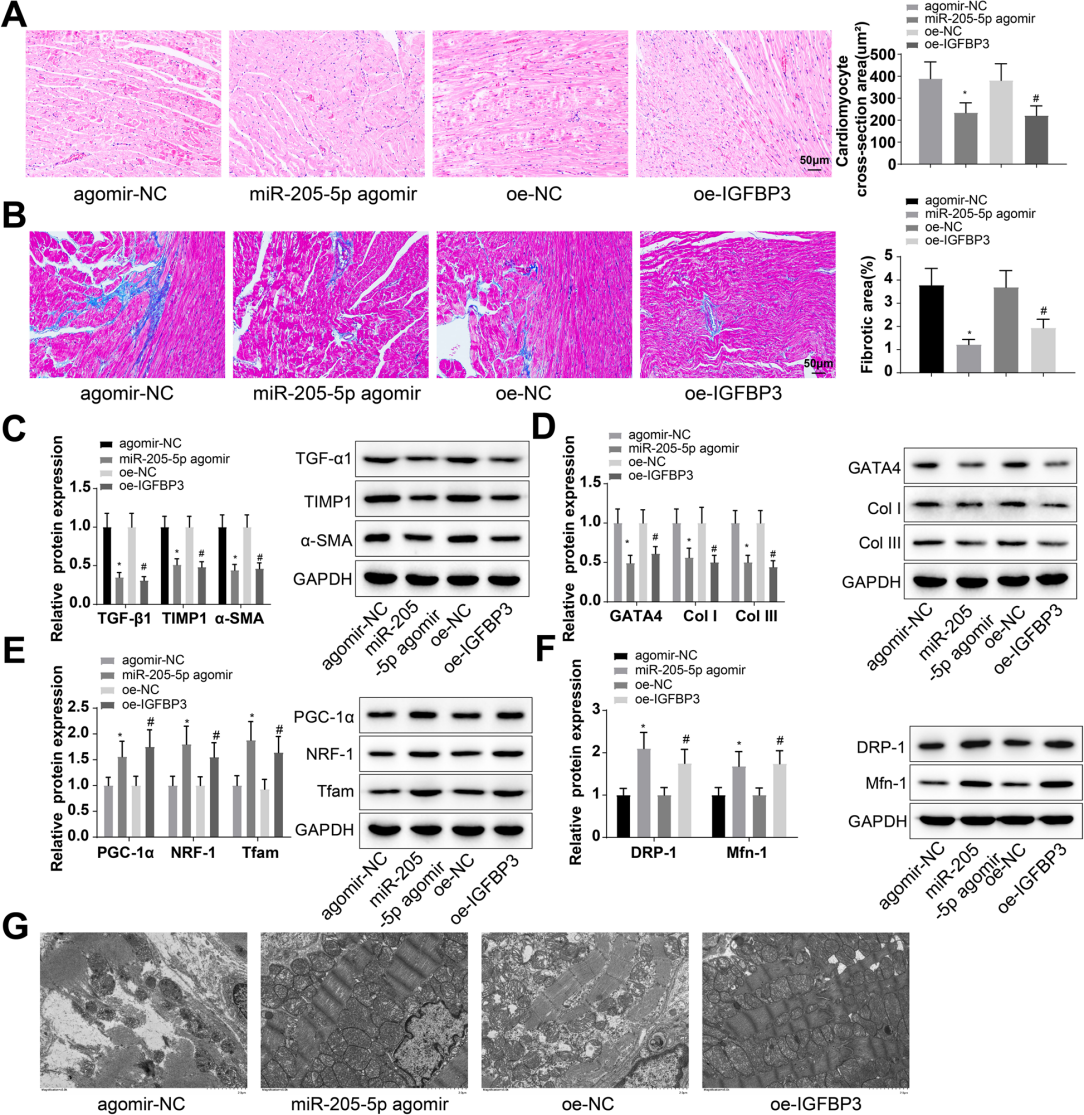
Upregulation of miR-205-5p or IGFBP3 attenuates the atrial fibrosis and mitochondrial damage caused by HFD. **A** Pathology of the left atrial tissues in mice injected with miR-205-5p agomir and oe-IGFBP3 was observed by H&E staining. **B** Masson staining was conducted for observing collagen deposition and atrial fibrosis in mice injected with miR-205-5p agomir and oe-IGFBP3. **C**, **D** Western blot analysis was conducted to examine the protein expression of key regulatory proteins of fibrosis (TGF-β1, TIMP1, and α-SMA) and hypertrophic markers (GATA4, Col I, and COL III) in mice injected with miR-205-5p agomir and oe-IGFBP3. **E**, **F** Western blot analysis was performed to examine the expression levels of mitochondrial biogenesis-related, division-related, and fusion-related proteins in the mouse atrial tissues. **G** The ultrastructure of the mitochondria in the left atrial tissues was visualized by a TEM. *p < 0.05, vs. the agomir-NC group; #*p* < 0.05, vs. the oe-NC group

H&E staining showed that the mice injected with miR-205-5p agomir and oe-IGFBP3 exhibited alleviated atrial myocardial arrangement and inflammasome infiltration (Fig. 6A). The findings of Masson staining showed a significant reduction in collagen deposition and improved fibrosis in mice injected with miR-205-5p agomir and oe-IGFBP3 (Fig. 6B). Additionally, TGF-β1, TIMP1, α-SMA, GATA4, Col I, and COL III expression levels were downregulated 0.35-fold, 0.51-fold, 0.44-fold, 0.49-fold, 0.56-fold, and 0.50-fold, respectively, in mice injected with miR-205-5p agomir (Fig. 6C, D). Downregulation of TGF-β1 (0.31-fold), TIMP1 (0.48-fold), α-SMA (0.46-fold), GATA4 (0.61-fold), Col I (0.50-fold), and COL III (0.44-fold) was also observed in mice injected with oe-IGFBP3 (Fig. 6C, D).

Furthermore, western blot analysis was performed to examine the expression levels of proteins related to mitochondrial biogenesis, division, and fusion in the mouse atrial tissues, and the ultrastructure of the mitochondria in the left atrial tissues was visualized by a TEM. The findings demonstrated that the expression levels of PGC-1, NRF-1, Tfam, DRP-1, and Mfn-1 were elevated 1.56-fold, 1.80-fold, 1.88-fold, 2.10-fold, and 1.68-fold, respectively, and the mitochondrial damage was attenuated in mice injected with miR-205-5p agomir (Fig. 6E–G). The expression levels of PGC-1α, NRF-1, Tfam, DRP-1, and Mfn-1 were elevated 1.75-fold, 1.55-fold, 1.76-fold, 1.75-fold, and 1.74-fold, respectively, and the mitochondrial damage was attenuated in mice injected with oe-IGFBP3 (Fig. 6E–G). The above results indicate that the upregulation of miR-205-5p or IGFBP3 ameliorates the atrial fibrosis and mitochondrial damage caused by HFD.

### Upregulation of miR-205-5p or IGFBP3 attenuates the atrial metabolic disorder and inflammatory response induced by HFD

**Fig. 7:**
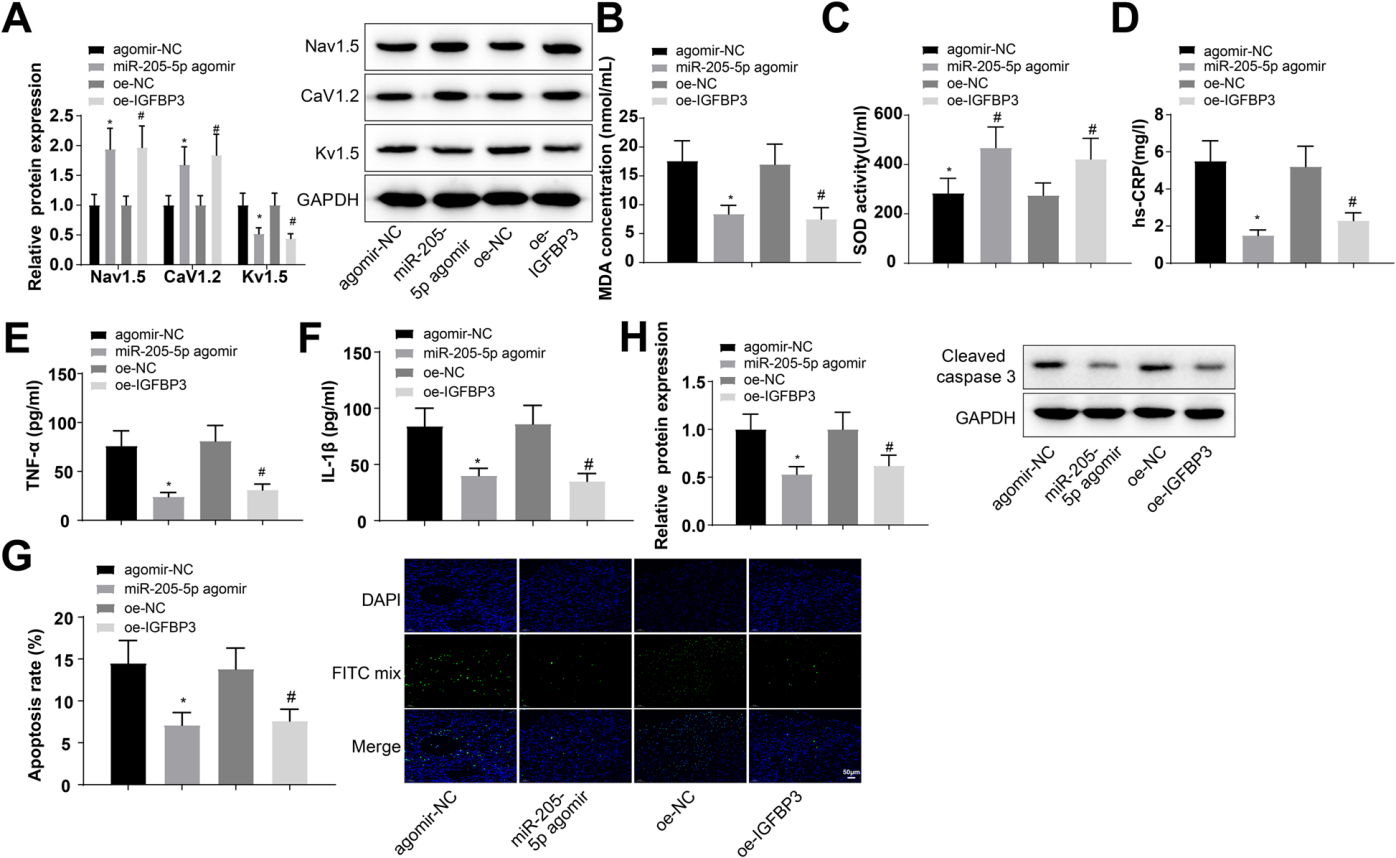
Upregulation of miR-205-5p or IGFBP3 attenuates the atrial metabolic disorder and inflammatory response induced by HFD. **A** The ionophoric proteins Nav1.5, CaV1.2, and Kv1.5 in mouse left atrial tissues were examined by western blot analysis. **B**, **C**. The levels of oxidative stress indicators MDA and SOD in atrial tissues were measured by ELISA. **D**–**F**. The expression levels of hs-CRP, TNF-α, and IL-1β in the left atrial tissues of mice in each group were measured by ELISA. **G** the tissue apoptosis was detected by TUNEL staining. **H** Expression of the key apoptotic enzyme caspase 3 was determined by western blot analysis. *p < 0.05, vs. the agomir-NC group; #*p* < 0.05 vs. the oe-NC group

It is reported that diet-induced obesity can lead to metabolic disorders in the body, accompanied by chronic inflammation [41]. We therefore explored the effects of miR-205-5p and IGFBP3 on HFD-induced atrial metabolism, oxidative stress, and inflammation. First, the ionophoric proteins Nav1.5, CaV1.2, and Kv1.5 in mouse left atrial tissues were examined by western blot analysis. We found that Nav1.5 and CaV1.2 expression levels were increased 1.94-fold and 1.68-fold, respectively, and Kv1.5 expression was decreased 0.52-fold in mice injected with miR-205-5p agomir (Fig. 7A). Nav1.5 and CaV1.2 expression levels were increased 1.97-fold and 1.84-fold, respectively, and Kv1.5 expression was decreased 0.44-fold in mice injected with oe-IGFBP3 (Fig. 7A).

Next, the levels of oxidative stress indicators MDA and SOD in atrial tissues were measured by ELISA, and the results showed decreased MDA contents (0.47-fold, 0.44-fold) and increased SOD contents (1.65-fold, 1.53-fold) in mice injected with miR-205-5p agomir and oe-IGFBP3 (Fig. 7B, C).

Furthermore, the expression levels of hs-CRP, TNF-α, and IL-1β in the left atrial tissues of mice in each group were measured by ELISA, with the apoptosis detected by TUNEL staining and the expression of the key apoptotic enzyme caspase 3 determined by western blot analysis. It was found that the expression levels of hs-CRP, TNF-α, and IL-1β were decreased 0.28-fold, 0.32-fold, and 0.48-fold, respectively, and the apoptosis index and cleaved caspase 3 expression were reduced 0.49-fold and 0.53-fold, respectively, in mice injected with miR-205-5p agomir (Fig. 7D–H). The expression levels of hs-CRP, TNF-α, and IL-1β were decreased 0.44-fold, 0.38-fold, and 0.41-fold, respectively, and the apoptosis index and cleaved caspase 3 expression were reduced 0.55-fold and 0.62-fold, respectively, in mice injected with oe-IGFBP3 (Fig. 7D–H). The aforesaid results reveal that upregulation of miR-205-5p or IGFBP3 can alleviate HFD-induced abnormalities in atrial ion channels, oxidative stress, inflammatory response, and apoptosis.

## Discussion

Despite great advancements over the past few decades, the fundamental mechanisms of maintaining AF have yet to be deciphered, and effective therapies for combating AF remain to be developed [42, 43]. MiRNAs have emerged as biomarkers for the diagnosis of AF and miRNA mimics or anti-miRNAs have been proposed as a therapeutic strategy to ameliorate this arrhythmic event [44, 45]. In this study, experiments were mainly conducted to assess the function and mechanism of miR-205-5p in the pathogenesis of AF. The main data in the present study expounded that re-expression of miR-205-5p, a downregulated miRNA in AF, could alleviate the fibrosis of atrial muscle cells and mouse atrial fibrosis, whose effect was potentially achieved through impairing EHMT2-dependent H3 histone methylation of IGFBP3.

The first finding observed in the study was that miR-205-5p expression was poor in the AF patients. Through the in vitro model of PA-induced atrial fibrosis, we found that overexpression of miR-205-5p acted to suppress the fibrosis of atrial muscle cells by reducing the levels of fibrotic markers (α-SMA, Col I, Col III). Tachycardia-evoked atrial fibrosis is an emblem of AF-related structural remodeling [46]. Fibrosis is characterized by immoderate synthesis and accumulation of extracellular matrix, supporting the functions of parenchymal cells with a surrounding network [47]. Recent studies have identified miR-205 as a protective target for the disorders in the cardiovascular system. For instance, enforced expression of miR-205-3p confers the anti-fibrotic effect on tanshinone IIA and its improving effect on ventricular remodeling post myocardial infarction [48]. Another study has uncovered the relation of miR-205-3p inhibition to particulate matte-elicited myocardial toxicity [49]. Myolysis, myocardial apoptosis, and activated fibrotic markers by fibroblasts and TGF-β are also modulated via inflammatory pathways, which can all lead to structural remodeling of the atria and AF [50]. Additionally, miR-205-5p exerts anti-inflammatory role in the course of lung damage following hip fracture through meditating the release of pro-inflammatory cytokines [51]. Our study showed consistent findings that miR-205-5p gain-of-function downregulated the pro-inflammatory TGF-β1, TNF-α, and IL-1β levels, supporting its protective effect against AF-induced damage. For validation, we developed a murine model of atrial fibrosis triggered by HFD. Through this model, miR-205-5p agomir elevated the expression of miR-205-5p and consequently repressed the atrial fibrosis, inflammasome infiltration, and mitochondria damage. A prior study has linked the elevation of CRP and IL-6 to significant inflammatory infiltrates, myocyte necrosis, and atrial fibrosis, contributing to incidence and persistence of AF [52]. Mitochondrial function is related to the generation of reactive oxygen species (ROS), control of calcium homeostasis, and change of oxygen consumption, which are crucial pathophysiological events pertaining to AF [53]. Elevation of miR-205-5p was unveiled in this study to reverse the HFD-induced abnormal atrial ion channels, oxidative stress, inflammation, and apoptosis, contributing to protection against AF.

Furthermore, this study offered evidence for the binding of miR-205-5p to EHMT2. Meanwhile, EHMT2 could elevate the enrichment of H3K9me2 but reduce the enrichment of H3K4me3 in the IGFBP3 promoter region. Either overexpression of EHMT2 or knockdown of IGFBP3 was substantiated to reverse the anti-fibrotic effect of miR-205-5p on atrial muscle cells, which is rarely mentioned in the previous studies. It is well-known that EHMT2 correlates with gene silencing in an H3K9me2-dependent fashion and that EHMT2 primarily functions to induce transcriptional repression by recruiting H3K9me2-binding proteins [54]. Exogenous IGFBP3 delivery may alleviate antigen-caused airway inflammation and hyperreactivity in mice with allergic airway disease [55]. A subsequent study also reveals the anti-inflammatory activity of IGFBP3 in the airway through its receptor-mediated caspase activation [56]. Also, the anti-inflammatory role of IGFBP3 is manifested with suppressed secretion of inflammatory cytokines (COX-2, IL-1β, and TNF-α) as well as reduced ROS activity [57]. Meanwhile, IGFBP-3 can suppress the release of TNF-α whereby restraining the apoptotic potential of retinal endothelial cells [58]. In keeping with the aforementioned findings, IGFBP3 overexpression was demonstrated to ameliorate the abnormalities in atrial ion channels, prevent the oxidative stress, and suppress the inflammation and apoptosis triggered by HFD in mice.

Currently, the HFD rodent model is considered to be one of the most widely used animal models to promote the treatment of diabetes, obesity, and related metabolic syndromes [59]. Nevertheless, the HFD model has limitations. Humans and mice respond differently to HFD and thus the HFD mouse model may not fully mimic the pathogenesis of atrial fibrosis in humans. Moreover, large animal experiments may be required to study if there is a direct relationship between HFD and the etiology of AF.

In summary, miR-205-5p acted as an anti-fibrotic miRNA in the context of AF by blocking the EHMT2-dependent IGFBP3 inhibition. The animal experimental data highlighted the significance of miR-205-5p agomir for combating AF for future clinical application. Moreover, recombinant human IGFBP3 has been testified to attenuate lipopolysaccharide-evoked acute lung inflammation through downregulating the levels of inflammation-promoting TNF-α, IL-6, and IL-1β [60]. It is intriguing to examine in the following days whether recombinant human IGFBP3 also works to AF-related inflammation in animal models before its practice in clinical trials. Since the anti-inflammation action of IGFBP3 is possibly attributed to its potential of inhibiting nuclear factor-κB (NF-κB) [57], further investigation on the interaction between IGFBP3 and NF-κB in the context of AF is warranted to offer a molecular basis for the mechanisms in the pathogenesis of AF.

## Data Availability

The datasets used or analyzed during the current study are available from the corresponding author on reasonable request.
